# Sodium nitrite reduces circulating asymmetric dimethylarginine in a reduced uterine perfusion pressure rat model of preeclampsia

**DOI:** 10.3389/fphar.2026.1939792

**Published:** 2026-09-18

**Authors:** Lucas C. da Costa, Maria Luiza S. Silva, Cristal J. Toghi, Aurélio H. Doreto, Hélio Kushima, Flávia Bessi Constantino Colenci, Carlos Alan Dias-Junior

**Affiliations:** Cardiovascular Pharmacology Laboratory, Department of Biophysics and Pharmacology, Institute of Biosciences, São Paulo State University, Botucatu, Brazil

**Keywords:** asymmetric dimethylarginine, endothelial dysfunction, nitric oxide, placental ischemia, Preeclampsia, sodium nitrite

## Abstract

**Introduction:**

Preeclampsia (PE) is characterized by hypertension, endothelial dysfunction, and reduced nitric oxide (NO) bioavailability. Asymmetric dimethylarginine (ADMA), an endogenous inhibitor of endothelial NO synthase, is elevated in PE and contributes to vascular dysfunction. NO may regulate ADMA production. NO formation from nitrite occurs under hypoxic-ischemic conditions. Thus, this study investigated whether sodium nitrite attenuates circulating ADMA levels and vascular endothelial dysfunction in placental ischemia induced by reduced uteroplacental perfusion pressure (RUPP) in pregnant rats.

**Methods:**

Pregnant Wistar rats were assigned to Sham, RUPP, Sham-Nitrite, and RUPP-Nitrite groups. Sodium nitrite (15 mg/kg/day) was administered orally from gestational day 14–21.

**Results:**

RUPP rats exhibited increased systolic, diastolic, and mean arterial pressure, reduced fetal and placental weights, impaired acetylcholine-induced relaxation, enhanced phenylephrine-induced vasoconstriction, and increased plasma ADMA levels compared to Sham group. Sodium nitrite treatment attenuated hypertension, improved fetal and placental parameters, partially restored endothelial function and reduced vascular hyper-reactivity. Plasma NO metabolites in the RUPP-Nitrite group were not significantly different from Sham. Importantly, sodium nitrite markedly attenuated the RUPP-induced increases in circulating ADMA, suggesting modulation of the ADMA-NO pathway in experimental PE.

**Discussion:**

These findings indicate that nitrite treatment may attenuate endothelial dysfunction and vascular impairment associated with placental ischemia, potentially involving modulation of circulating ADMA.

## 1 Introduction

PE, a hypertensive disorder specific to pregnancy, according to the American College of Obstetricians and Gynecologists (ACOG), is characterized by the onset of hypertension after the 20th gestational week associated with proteinuria or maternal organ injury (American College of Obstetricians and Gynecologists, 2020). PE may also occur superimposed on chronic hypertension, when proteinuria, thrombocytopenia, or other systemic manifestations of the disease develop (Brown et al., 2018). In a severe stage, termed eclampsia, vascular endothelial impairment in the central nervous system may occur, potentially progressing to tonic–clonic seizures (Hutcheon et al., 2011).

PE affects approximately 2%–8% of pregnancies worldwide, with an average prevalence of approximately 4.4% (Vera-Ponce et al., 2025). Despite advances in understanding the pathophysiology of PE, currently available therapeutic options remain limited and, in many cases, essentially palliative (Rosenberg and Seely, 2024). Current clinical management is primarily based on blood pressure control and prevention of maternal and fetal complications, with delivery remaining the only definitive treatment for PE (Rosenberg and Seely, 2024).

The pathophysiology of PE is mainly associated with placental insufficiency, in which disrupted interactions between the decidua and trophoblast compromise uteroplacental circulation and development from the first trimester of pregnancy (Guerby et al., 2021). Placental development in a pathophysiologically hypoxic environment promotes increased oxidative stress, which progressively stimulates the release of apoptotic and necrotic trophoblastic fragments, pro-inflammatory cytokines, and antiangiogenic factors into the maternal circulation (Redman and Sargent, 2005; Burton et al., 2019). These factors lead to systemic endothelial dysfunction, which is mainly manifested by hypertension and proteinuria (Brown et al., 2018). Although PE originates in the placenta, it markedly impairs the maternal endothelium and has been related to alterations in several markers of endothelial activation and dysfunction (Guerby et al., 2021; Wang et al., 2009). In this context, plasma levels of asymmetric dimethylarginine (ADMA), an endogenous inhibitor of endothelial nitric oxide (NO) synthesis, have been found to be significantly elevated in women with PE compared with normotensive pregnant women (Németh et al., 2018). Thus, increased ADMA contributes to reducing NO bioavailability and, consequently, to the endothelial dysfunction observed in PE (Németh et al., 2018).

ADMA is an endogenous arginine derivative produced by the methylation of arginine residues in proteins and subsequently released into the plasma (Leone et al., 1992). ADMA acts as a competitive inhibitor of endothelial NO synthase (eNOS), reducing the conversion of L-arginine into NO. NO is a crucial vasodilator for vascular homeostasis and the regulation of endothelial function, including arterial relaxation and placental perfusion during pregnancy (Boeger, 2003). Importantly, cohort and prospective investigations have demonstrated elevated ADMA levels before the onset of clinical symptoms of PE, suggesting its early involvement in the pathophysiology of this syndrome (Savvidou et al., 2003). Furthermore, experimental models and systematic reviews have highlighted the role of ADMA in inhibiting NO synthesis and promoting vasoconstriction and endothelial dysfunction, which are central mechanisms in PE (Speer et al., 2008; Mao et al., 2009; Dymara-Konopka and Laskowska, 2019). Interestingly, recent *in vitro* studies have demonstrated that cellular ADMA production may be regulated by endogenous NO (Taniguchi et al., 2024).

Unlike the classical eNOS-dependent pathway, which requires molecular oxygen, NO may also be generated by an alternative pathway based on nitrite (NO_2_^−^) reduction, which is particularly relevant under hypoxic conditions (Lundberg et al., 2008) and remains incompletely understood in PE. NO_2_^−^, previously considered an inert metabolite of NO, is currently recognized as an important circulating reservoir of NO, which can be reduced to NO through different enzymatic and non-enzymatic mechanisms, particularly in low-oxygen environments (Lundberg et al., 2008). Recent clinical investigation has shown that circulating NO_2_^−^ levels are reduced in women with PE compared with normotensive pregnant women, reflecting lower NO_2_^−^ bioavailability as an alternative NO precursor and contributing to the endothelial dysfunction characteristic of the disease (Possomato‐Vieira et al., 2021). Therefore, the main objective of the present study was to investigate the effects of sodium nitrite treatment on circulating ADMA levels in normotensive pregnant rats and preeclamptic-like rats, as well as on hemodynamic changes, fetal and placental parameters, vascular endothelial function, and plasma NO metabolites.

## 2 Materials and methods

### 2.1 Animals

Male and female Wistar rats were used in this study. Mating was performed by housing two females with one male overnight. On the following morning, vaginal smears were collected from the females to detect the presence of spermatozoa. The observation of spermatozoa was considered a positive indicator of pregnancy, and this day was designated as gestational day 1.

Pregnant rats were weighed and randomly assigned to the experimental groups. The experimental protocol (No. 6707090320) was approved on 30 March 2020, by the Animal Ethics Committee of the Institute of Biosciences of Botucatu, Sao Paulo State University. All procedures were conducted in accordance with the ARRIVE (Animal Research: Reporting of *In Vivo* Experiments) guidelines.

### 2.2 Surgical procedure for reduction of uteroplacental perfusion pressure (RUPP)

Experimental PE was induced using the RUPP surgical model on gestational day 14. The surgical procedure was performed as follows: anesthesia with isoflurane (1.5%–2%), ventral midline incision, placement of a 0.203 mm silver clip on the lower abdominal aorta (above the iliac bifurcation), placement of a 0.100 mm silver clip on the right uterine artery branch, placement of a 0.100 mm silver clip on the left uterine artery branch, and closure of the ventral midline incision (Martins et al., 2023; Gilbert et al., 2007). The Sham and Sham-Nitrite groups were also anesthetized with isoflurane (1.5%–2%) and underwent a sham surgical procedure on gestational day 14, consisting of a ventral midline incision and subsequent suturing without clip placement.

### 2.3 Experimental groups

The experimental groups consisted of pregnant rats of the same age (90 days old), randomly divided into four groups (n = 10 per group) as follows: group 1 – Sham: pregnant rats subjected to a sham surgical procedure on gestational day 14 without any interference in blood flow and treated with saline solution from gestational day 14 to day 21; group 2 – RUPP: pregnant rats subjected to the RUPP surgical procedure on gestational day 14 to induce clinical manifestations of PE and treated with saline solution from gestational day 14 to day 21; group 3 – Sham-Nitrite: pregnant rats subjected to a sham surgical procedure on gestational day 14 without any interference in blood flow and treated with sodium nitrite solution from gestational day 14 to day 21; group 4 – RUPP-Nitrite: pregnant rats subjected to the RUPP surgical procedure to induce clinical manifestations of PE and treated with sodium nitrite solution from gestational day 14 to day 21.

Sodium nitrite (NaNO_2_^−^; S2252; Sigma-Aldrich, St. Louis, MO, USA) was administered by oral gavage at a dose of 15 mg/kg/day, once daily, from gestational day 14 to day 21. The dose of sodium nitrite was chosen based on previous studies showing that this dose exerts relevant antihypertensive effects without observing adverse toxicological effects in rats (Amaral et al., 2015; Classen et al., 1990; Pinheiro et al., 2014; Pinheiro et al., 2015). Control groups received an equivalent volume of saline solution (0.9% NaCl) as vehicle. Drug solutions were prepared daily and administered immediately, as previously standardized (Martins et al., 2023).

### 2.4 Measurement of maternal hemodynamic parameters

A polyethylene catheter (#2270835, Thermo Fisher Scientific, Waltham, MA, USA) was inserted into the carotid artery, and its distal end was exteriorized at the posterior region of the neck following anesthesia with isoflurane on gestational day 21. To allow the rats to recover from anesthesia, at least 1 hour passed, and observations were made until each rat showed no signs of pain. After the recovery, the carotid arterial catheter was connected to a pressure transducer coupled to an amplifier and data acquisition system (MP150CE, Biopac Systems Inc., Goleta, CA, USA). Systolic, diastolic, and mean arterial pressure, as well as heart rate, were continuously recorded for 20 min following a stabilization period in conscious, freely moving rats using AcqKnowledge software version 3.2.1 (Biopac Systems Inc., Goleta, CA, USA) (da Silva et al., 2026).

### 2.5 Euthanasia and tissue collection

On gestational day 21, the rats were anesthetized with isoflurane (3%) via inhalation. After loss of sensory and motor reflexes, cardiac puncture was performed for blood collection and euthanasia by exsanguination. Subsequently, each female underwent a cesarean section for collection of placentas, fetuses, and thoracic artery.

For plasma separation, blood samples were stored in lyophilized tubes containing heparin (Vacutainer, Becton, Dickinson, Oxford, UK), centrifuged at 4 °C (for 15 min) at 2,000 RPM, and stored at −80 °C until biochemical analyses.

For evaluation of endothelial and vascular function through vascular reactivity, the thoracic aorta was removed and maintained in cold Krebs–Henseleit solution.

### 2.6 Placental and fetal parameters

Litter size was expressed as the total number of fetuses per litter, while fetal and placental weights were expressed in grams (g) and calculated as the mean weight per litter. The litter was considered the experimental unit, with “n” representing the number of dams per experimental group. Individual fetuses or placentas were not treated as independent observations.

### 2.7 Evaluation of endothelial and vascular function

The thoracic aorta of each rat was dissected and sectioned into 2 mm-length rings, with arteries examined both with intact endothelium and without endothelium. The aortic rings were suspended between two tungsten hooks (40 µm in diameter) inserted through the lumen. One hook was fixed, and the other connected to a force transducer (FORT10, Transbridge 4M, World Precision Instruments, WPI, Sarasota, FL, USA). The preparations were placed in organ bath chambers at 37 °C containing Krebs–Henseleit solution (composition in mmol/L: NaCl 130; KCl 4.7; CaCl_2_ 1.6; KH_2_PO_4_ 1.2; MgSO_4_ 1.2; NaHCO_3_ 15; glucose 11.1), pH 7.4, continuously bubbled with a carbogen mixture (95% O_2_ and 5% CO_2_). After 30 min, vascular tension was equilibrated at a resting tension of 1.5 g. The arterial segments were contracted with potassium chloride (KCl, 96 mM) to assess vascular smooth muscle viability (Martins et al., 2023).

Endothelial integrity was evaluated based on the response after pre-contraction with phenylephrine (Phe; 10^−6^ M), followed by cumulative concentrations of acetylcholine (ACh; 10^−9^ to 10^−4^ M). Rings that exhibited less than 80% relaxation of Phe-induced contraction were excluded. Subsequently, arterial segments were washed with Krebs–Henseleit solution (3–5 times) and challenged with increasing concentrations of Phe (10^−10^ to 10^−4^ M) to generate concentration–response curves.

The role of NO in endothelium-dependent vascular relaxation was assessed using concentration–response curves to ACh in the presence of the non-selective NO synthase inhibitor Nω-nitro-L-arginine methyl ester (L-NAME; N5751, Sigma-Aldrich, St. Louis, MO, USA, 10^−3^ M), as previously described (da Silva et al., 2026).

To assess endothelium-independent NO-induced vascular relaxation, aortic rings with intact or denuded endothelium were pre-contracted with Phe (10^−6^ M) and exposed to cumulative concentrations of sodium nitroprusside (SNP; 10^−11^ to 10^−6^ M). Concentration–response curves to SNP were generated to evaluate vascular smooth muscle responsiveness to an exogenous NO donor.

Concentration–response curves were constructed. ACh-induced responses were expressed as percentage (%) relaxation relative to Phe-induced contraction. SNP-induced responses were expressed as percentage (%) relaxation relative to the Phe-induced pre-contraction. Maximal responses to Phe were recorded and expressed in grams (g), as previously described (Martins et al., 2023; Teoh et al., 2000).

### 2.8 Determination of ADMA levels

Plasma concentrations of ADMA were determined by enzyme-linked immunosorbent assay (ELISA) using a commercial kit (#E-EL-0042, Elabscience Ltd., Wuhan, Hubei, China), following the manufacturer’s instructions. This ELISA kit demonstrated an intra-assay CV of <10% and an inter-assay CV of <12%. Briefly, the assay was performed in duplicate in 96-well microplates (Thermo Fisher Scientific, Waltham, MA, USA), using a standard curve for quantification. Absorbance was measured at 450 nm using a microplate spectrophotometer (Synergy 4, BioTek, Winooski, VT, USA), and concentrations were calculated using a four-parameter logistic (4-PL) regression model, as previously detected in rodents (da Silva et al., 2026; Schulze et al., 2004).

### 2.9 Determination of NO metabolites (NOx) in plasma

Plasma NOx levels were quantified using the Griess method. Briefly, samples (190 µL) were deproteinized using zinc acetate (10 μL, 300 g/L), followed by centrifugation at 10,000 × g (4 °C, for 30 min). For the reaction step, 50 µL of the supernatant were transferred to 96-well microplates (Thermo Fisher Scientific, Waltham, MA, USA), followed by the addition of 100 µL vanadium chloride III (5%, w/v; 208,272, Sigma-Aldrich, St. Louis, MO, USA), 50 µL sulfanilamide (2%, w/v; S9251, Sigma-Aldrich), and 50 μL N-(1-naphthyl)ethylenediamine dihydrochloride (NED, 0.1%, v/v; N9125, Sigma-Aldrich, St. Louis, MO, USA). The reaction mixture was maintained under constant agitation and protected from light in a Thermomixer (Eppendorf, Hamburg, Germany) for 3 h at 37 °C. A standard curve based on increasing concentrations of sodium nitrite (1.56–100 μmol/L; S2252, Sigma-Aldrich, St. Louis, MO, USA) was processed identically for calibration. Absorbance was measured at 535 nm using a Synergy four microplate reader (BioTek Instruments, Winooski, VT, USA), and results were expressed as µmol/L (Tatsch et al., 2011).

### 2.10 Statistical analysis

Statistical analyses were performed using GraphPad Prism® software (version 8.0.1, San Diego, CA, USA). Data normality was assessed using the Shapiro–Wilk test. For hemodynamic, fetal, placental, and biochemical parameters, two-way analysis of variance (ANOVA) followed by Tukey’s *post hoc* test was applied. Additionally, two-way repeated measures ANOVA with Tukey’s *post hoc* test was used to evaluate differences in vascular responses to ACh, Phe, and SNP (point-by-point analysis) in vascular reactivity experiments. Statistical significance was set at *p* < 0.05, and results were expressed as mean ± standard error of the mean (SEM). In Figures 1–6, statistically significant differences between respective groups are indicated by comparison brackets with *post hoc* symbols (*p < 0.05,**p < 0.01, and ***p < 0.001). In vascular reactivity graphs (Figures 7–9), *post hoc* symbols, with or without brackets, indicate statistically significant differences between groups at the corresponding concentrations.

**FIGURE 1:**
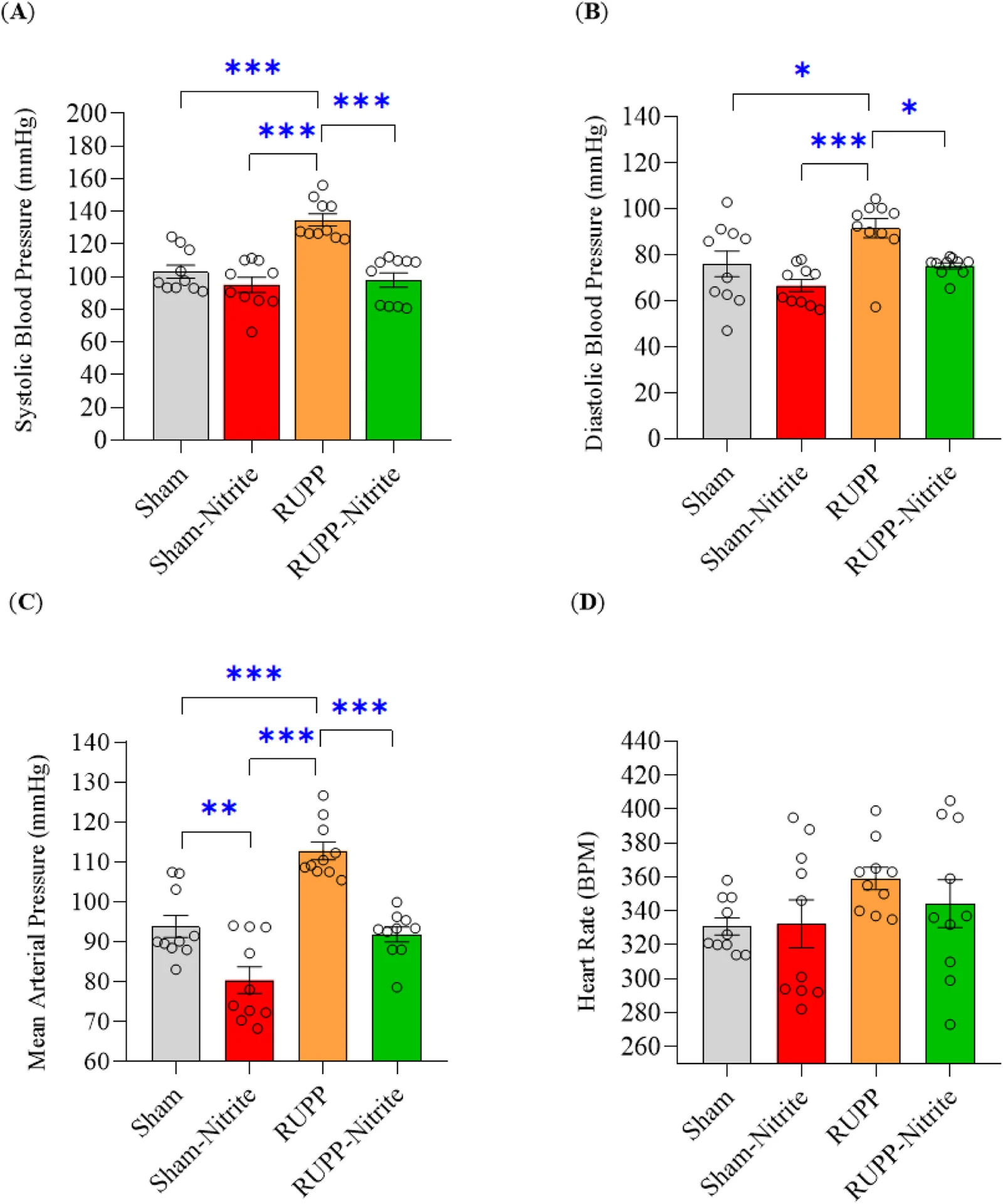
Effects of the sodium nitrate treatment on the mother’s systolic arterial pressure **(A)**, diastolic arterial pressure **(B)**, mean arterial pressure **(C)** and heart rate **(D)** measured on gestational day 21 in the Sham, Sham-Nitrite, RUPP, and RUPP-Nitrite groups (n = 10 animals per group). Data are expressed as mean ± SEM. Statistically significant differences between groups are indicated by comparison brackets with *post hoc* symbols based on two-way ANOVA followed by Tukey’s *post hoc* test. *p < 0.05, **p < 0.01, and ***p < 0.001.

## 3 Results

### 3.1 Effects of sodium nitrite on hemodynamic parameters

On gestational day 21, significant increases in systolic, diastolic and mean arterial pressures were observed in rats subjected to RUPP compared to the Sham, Sham-Nitrite, and RUPP-Nitrite groups (Figures 1A–C; Table 1). Sodium nitrite treatment in RUPP + Nitrite rats counteracted increases in systolic, diastolic, and mean arterial pressures (Figures 1A–C; Table 1). Significant decrease in mean arterial pressure was observed in the Sham-Nitrite group compared to the Sham group (Figure 1C; Table 1), and a trend toward increased heart rate was noted in the RUPP group compared to the Sham group (Figure 1D; Table 1).

**TABLE 1:** Hemodynamic, placental, fetal, biochemical, and vascular parameters from the Sham, Sham-Nitrite, RUPP, and RUPP-Nitrite groups.

Parameter	Sham	Sham-nitrite	RUPP	RUPP-nitrite	*p* interaction	*p* treatment effect	*p* surgery effect
Systolic blood pressure (mmHg)	103 ± 4	95 ± 4	134 ± 3	98 ± 4	**0.0017**	**<0.0001**	**0.0002**
Diastolic blood pressure (mmHg)	76 ± 5	66 ± 2	91 ± 4	75 ± 1	0.3616	**0.0016**	**0.0029**
Mean blood pressure (mmHg)	93 ± 2	80 ± 3	112 ± 2	91 ± 2	0.1595	**<0.0001**	**<0.0001**
Heart rate (bpm)	330 ± 5	332 ± 14	359 ± 6	344 ± 14	0.4511	0.5436	0.0700
Placental weight (g)	0.337 ± 0.009	0.345 ± 0.010	0.275 ± 0.008	0.330 ± 0.010	**0.0210**	**0.0026**	**0.0003**
Fetal weight (g)	4.14 ± 0.34	3.76 ± 0.24	3.14 ± 0.17	3.64 ± 0.22	0.0887	0.8152	**0.0348**
Litter size (number of fetuses)	13.30 ± 0.57	11.80 ± 0.44	6.10 ± 0.45	10.20 ± 0.57	**<0.0001**	**0.0165**	**<0.0001**
ADMA (pg/mL)	55 ± 5	65 ± 10	149 ± 12	68 ± 5	**<0.0001**	**0.0003**	**<0.0001**
Plasma NOx (mmol/L)	93 ± 3	99 ± 7	79 ± 1	88 ± 3	0.8027	0.1155	**0.0088**
ACh with intact endothelium E_*max*_ (% relaxation of phe-induced contraction)	83.40 ± 2.38	77.70 ± 3.45	53.02 ± 5.30	61.62 ± 5.18	0.1321	0.7546	**<0.0001**
ACh with intact endothelium pEC_50_ (−log M)	4.90 ± 0.09	5.16 ± 0.18	6.12 ± 0.27	5.80 ± 0.28	0.5309	0.0958	0.3250
Phe with intact endothelium E_*max*_ (g)	1.68 ± 0.08	1.57 ± 0.12	2.58 ± 0.15	1.87 ± 0.14	**0.0371**	**0.0038**	**<0.0001**
Phe with intact endothelium pEC_50_ (−log M)	6.90 ± 0.14	6.53 ± 0.14	6.72 ± 0.16	6.56 ± 0.10	**0.0311**	0.9184	0.2473
Phe with denuded endothelium E_*max*_ (g)	2.39 ± 0.30	2.10 ± 0.15	3.63 ± 0.51	2.47 ± 0.21	**0.0327**	**0.0006**	**0.0001**
Phe with denuded endothelium pEC_50_ (−log M)	7.05 ± 0.34	6.59 ± 0.18	7.80 ± 0.46	6.98 ± 0.23	0.1019	**0.0024**	0.2318
SNP with intact endothelium E_*max*_ (% relaxation of phe-induced contraction)	94.48 ± 1.10	92.76 ± 0.79	96.71 ± 0.86	94.12 ± 1.54	0.8059	0.0665	0.1075
SNP with intact endothelium pEC_50_ (−log M)	8.23 ± 0.22	8.22 ± 0.22	8.31 ± 0.15	8.36 ± 0.24	0.7326	0.8511	0.4076
SNP with denuded endothelium E_*max*_ (% relaxation of phe-induced contraction)	94.64 ± 1.07	95.34 ± 0.91	96.91 ± 1.10	94.20 ± 1.27	0.3125	0.3475	0.7021
SNP with denuded endothelium pEC_50_ (−log M)	8.33 ± 0.20	8.43 ± 0.06	8.76 ± 0.22	8.39 ± 0.07	0.3198	0.5161	0.2943

Data are presented as mean ± SEM, for Sham, Sham-Nitrite; RUPP, and RUPP-Nitrite groups (n = 10 animals per group). Statistical analysis was performed using two-way ANOVA, considering surgery (Sham vs. RUPP) and treatment (vehicle vs. Sodium nitrite) as factors. A value of *p* < 0.05 was considered statistically significant. Bold values are significant p-values. ACh—acetylcholine; ADMA—asymmetric dimethylarginine; NO—nitric oxide; NOx—nitric oxide metabolites; Phe—phenylephrine; RUPP—reduced uterine perfusion pressure; SNP—sodium nitroprusside.

### 3.2 Effects of sodium nitrite on placental weight

A significant decrease in placental weight was observed in the RUPP group compared to the Sham, Sham-Nitrite, and RUPP-Nitrite groups (Figure 2; Table 1). Sodium nitrite treatment in RUPP + Nitrite rats reversed decreases in placental weight (Figure 2; Table 1).

**FIGURE 2:**
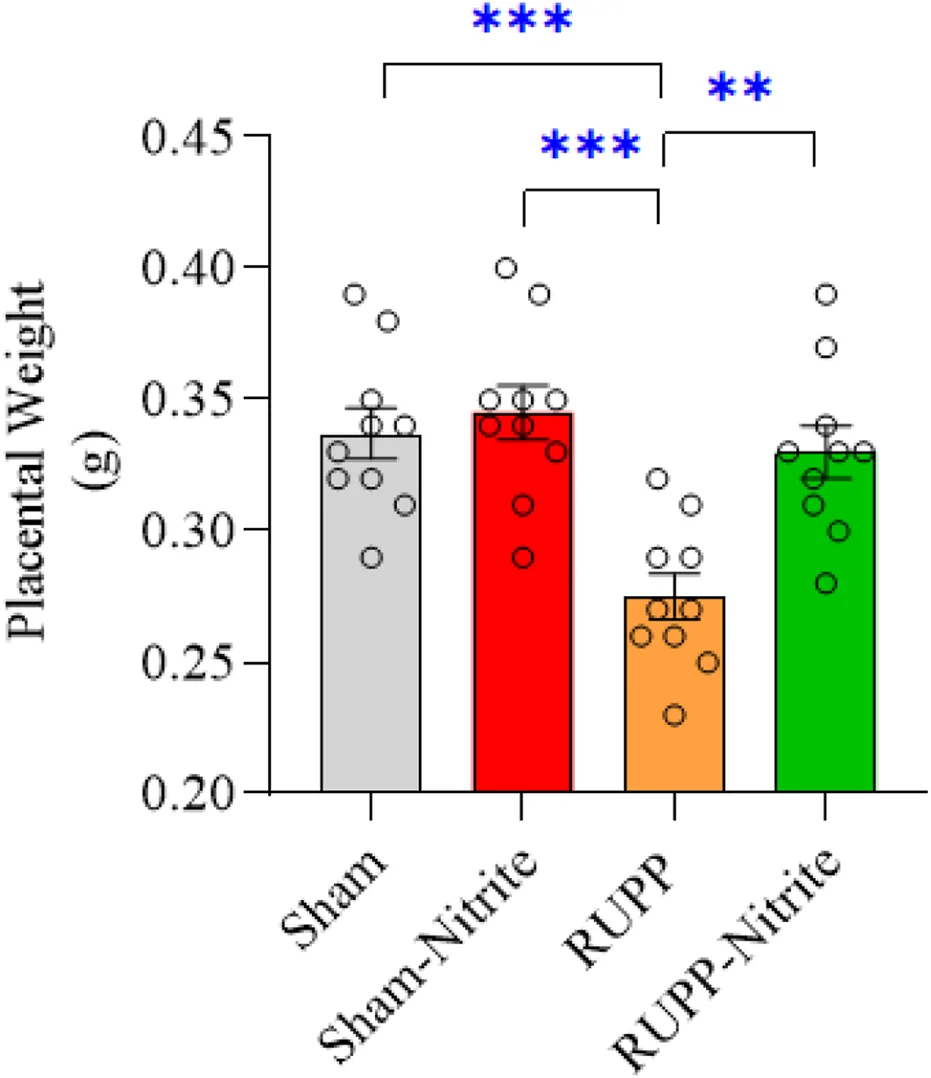
Effects of sodium nitrite treatment on placental weight registered on gestational day 21 in the Sham, Sham-Nitrite, RUPP, and RUPP-Nitrite groups (n = 10 animals per group). Data are expressed as mean ± SEM. Statistically significant differences between groups are indicated by comparison brackets with *post hoc* symbols based on two-way ANOVA followed by Tukey’s *post hoc* test. **p < 0.01 and ***p < 0.001.

### 3.3 Effects of sodium nitrite on fetal weight

Fetal weight was significantly lower in the RUPP group compared to the Sham, Sham-Nitrite, and RUPP-Nitrite groups (Figure 3; Table 1). However, no significant differences were observed between the Sham and RUPP-Nitrite groups, indicating that fetal weight in RUPP-Nitrite rats was not significantly reduced compared with the Sham group. (Figure 3; Table 1).

**FIGURE 3:**
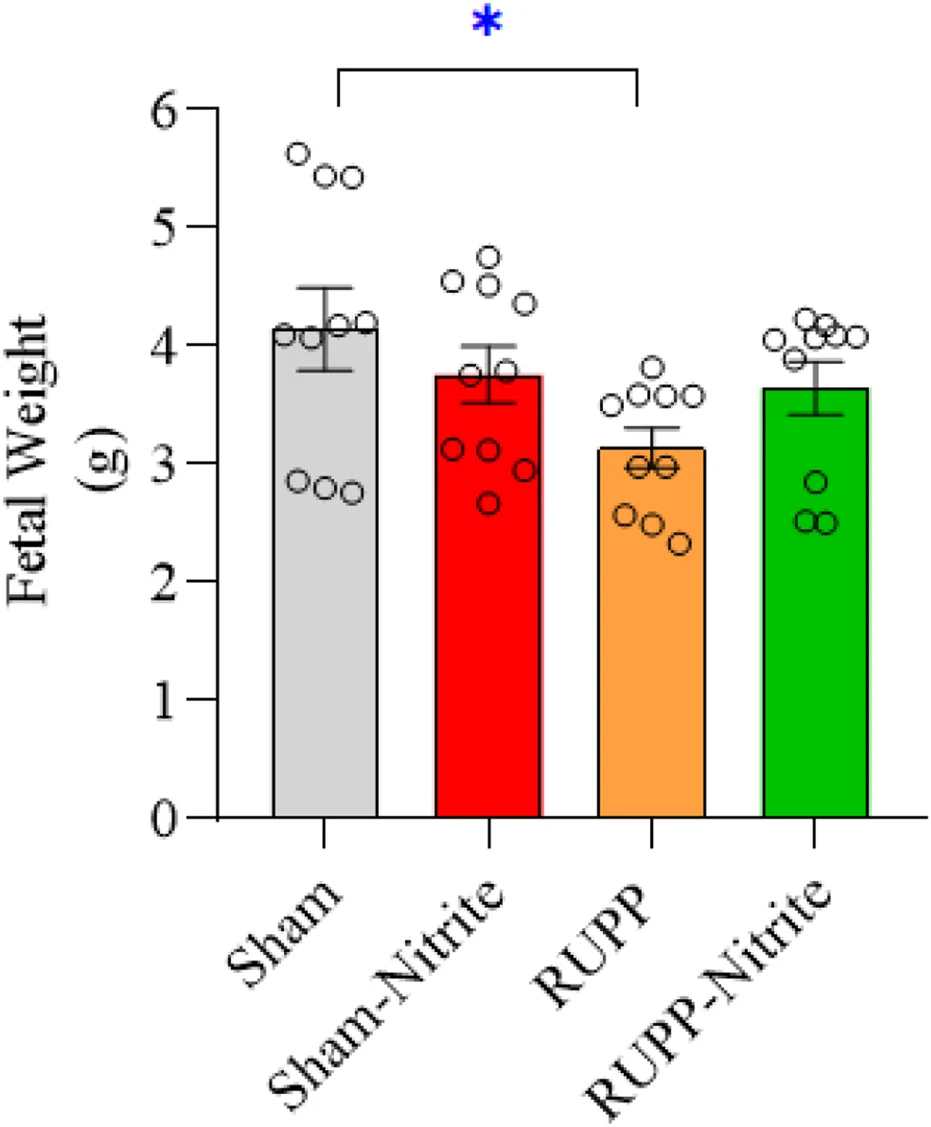
Effects of the sodium nitrite treatment on fetal weight recorded on gestational day 21 in the Sham, Sham-Nitrite, RUPP, and RUPP-Nitrite groups (n = 10 animals per group). Data are expressed as mean ± SEM. Statistically significant differences between groups are indicated by comparison brackets with *post hoc* symbols based on two-way ANOVA followed by Tukey’s *post hoc* test. *p < 0.05.

### 3.4 Effects of sodium nitrite on the total number of fetuses

Significant decreases in the number of fetuses per litter were observed in the RUPP group compared with Sham, Sham-Nitrite, and RUPP-Nitrite groups, and in the RUPP-Nitrite group compared with Sham group (Figure 4; Table 1). Sodium nitrite treatment in RUPP + Nitrite rats attenuated reductions in litter size (Figure 4; Table 1).

**FIGURE 4:**
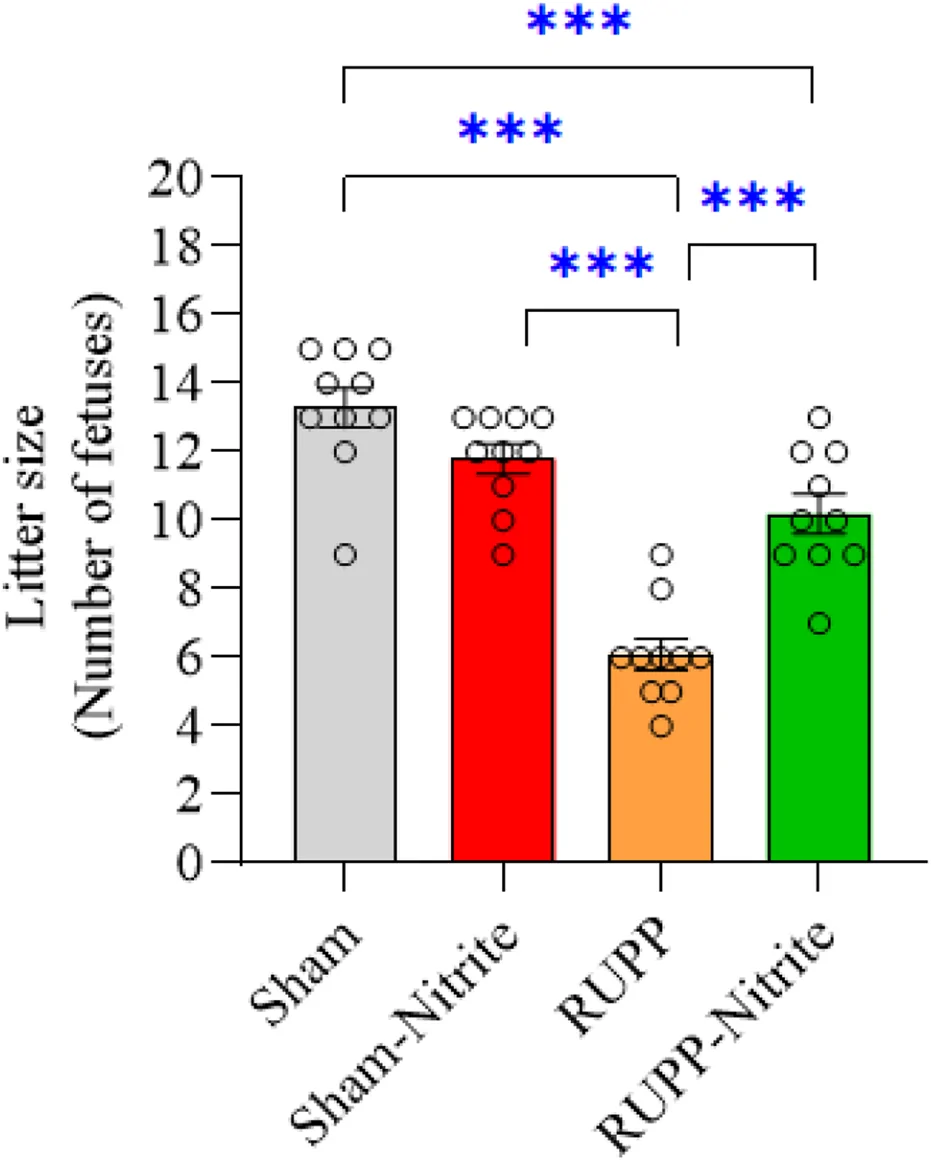
Effects of sodium nitrite treatment on the total number of fetuses per litter (Litter size) recorded on gestational day 21 in the Sham, Sham-Nitrite, RUPP, and RUPP-Nitrite groups (n = 10 animals per group). Data are expressed as mean ± SEM. Statistically significant differences between groups are indicated by comparison brackets with *post hoc* symbols based on two-way ANOVA followed by Tukey’s *post hoc* test. ***p < 0.001.

### 3.5 Effects of sodium nitrite on plasma ADMA levels

Significant increases in ADMA levels were observed in the RUPP group compared to the Sham, Sham-Nitrite, and RUPP-Nitrite groups (Figure 5; Table 1). Conversely, the Sham-Nitrite and RUPP-Nitrite groups displayed no significant differences relative to the Sham group (Figure 5; Table 1).

**FIGURE 5:**
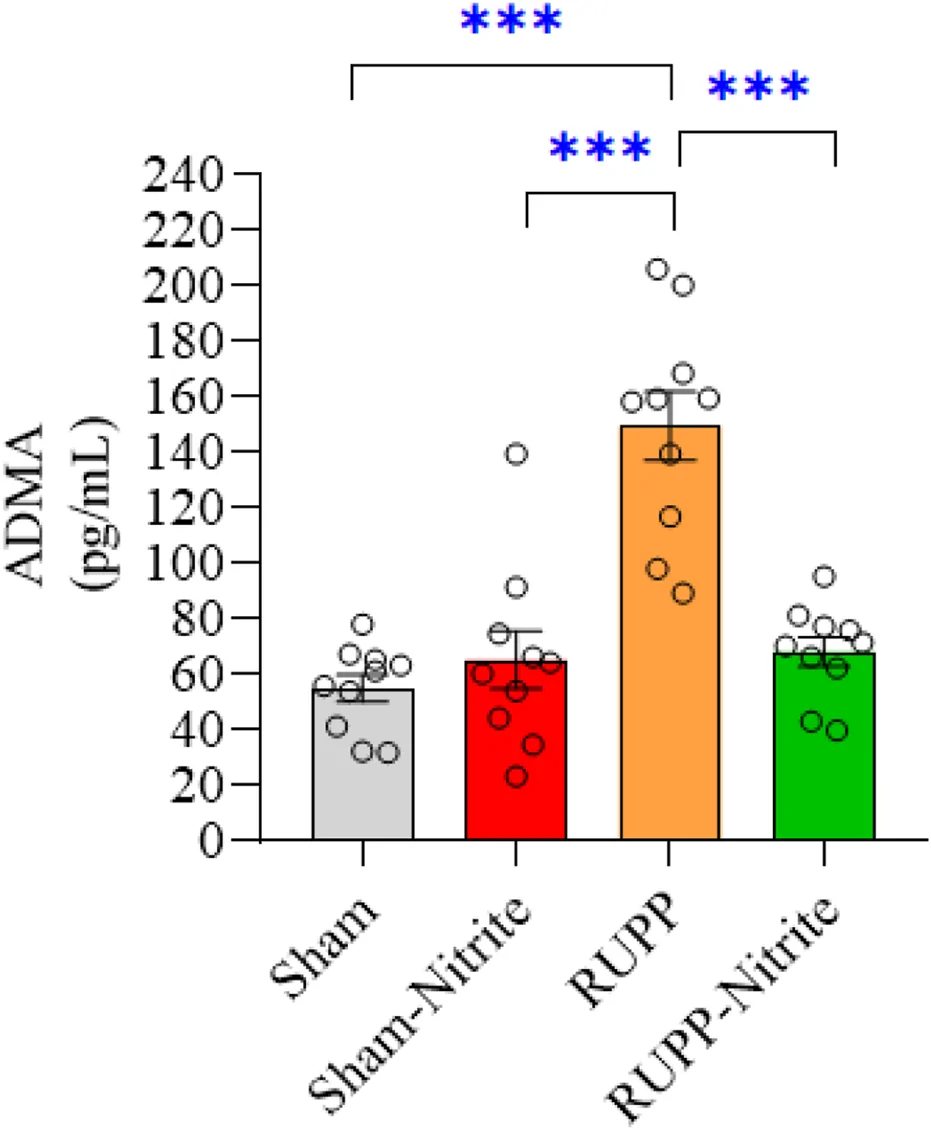
Effects of sodium nitrite treatment on Asymmetric Dimethylarginine (ADMA) levels measured on gestational day 21 in the Sham, Sham-Nitrite, RUPP, and RUPP-Nitrite groups (n = 10 animals per group). Data are expressed as mean ± SEM. Statistically significant differences between groups are indicated by comparison brackets with *post hoc* symbols based on two-way ANOVA followed by Tukey’s *post hoc* test. ***p < 0.001.

### 3.6 Effects of sodium nitrite on plasma NOx

A significant reduction in NOx was noted in the RUPP group compared to the Sham-Nitrite group, and a trend toward a reduction was observed in the RUPP group compared to the Sham group (Figure 6; Table 1), whilst the Sham-Nitrite and RUPP-Nitrite groups showed no significant differences compared to the Sham group (Figure 6; Table 1).

**FIGURE 6:**
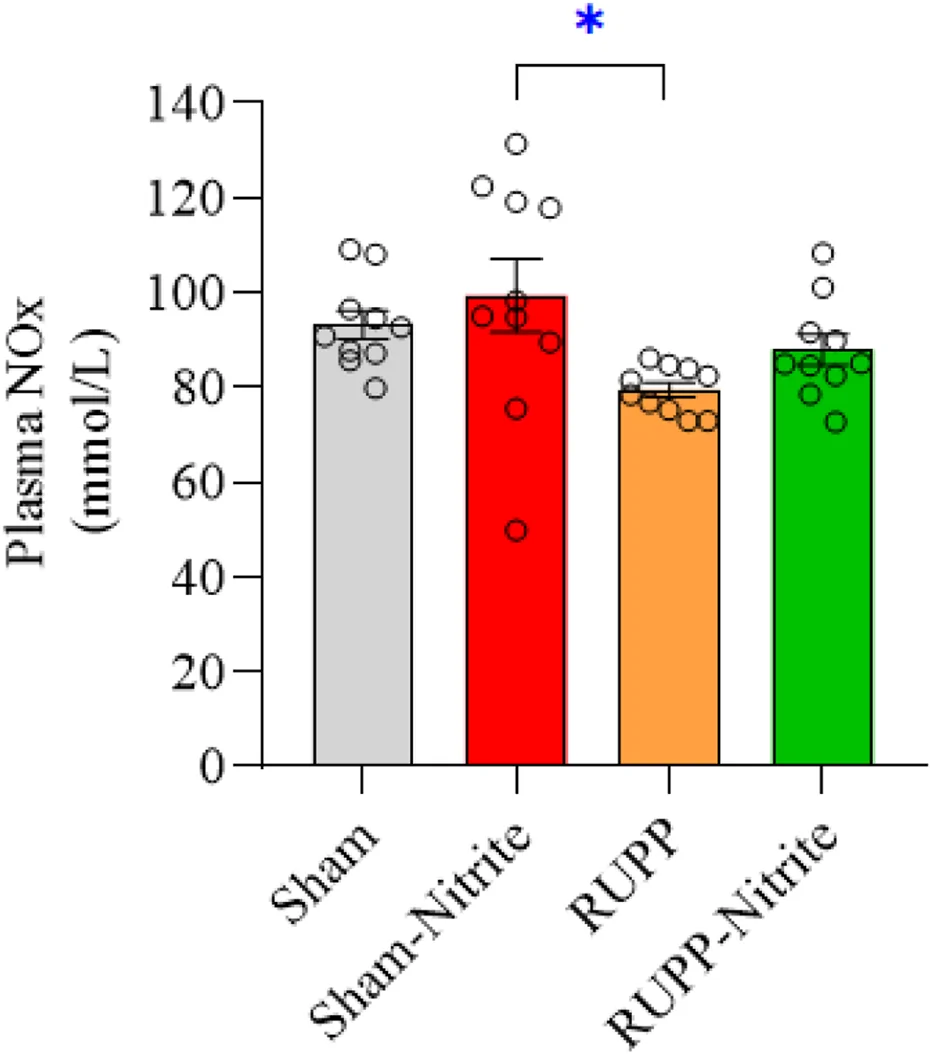
Effects of sodium nitrite treatment on Plasma NO metabolites (NOx) measured on gestational day 21 in the Sham, Sham-Nitrite, RUPP, and RUPP-Nitrite groups (n = 10 animals per group). Data are expressed as mean ± SEM. Statistically significant differences between groups are indicated by comparison brackets with *post hoc* symbols based on two-way ANOVA followed by Tukey’s *post hoc* test. *p < 0.05.

### 3.7 Effects of sodium nitrite on ACh-Induced vascular relaxation

Significant impairments in ACh-induced vascular relaxation were identified in endothelium-intact thoracic aortic rings pre-contracted with Phe in the RUPP group compared to the Sham group at concentrations of 10^−6^, 10^−5^, and 10^−4^ M; in the RUPP group compared to the Sham-Nitrite group at a concentration of 10^−6^ M; and in the RUPP-Nitrite group compared to the Sham group at concentrations of 10^−5^ and 10^−4^ M (Figure 7A; Table 1). In endothelium-denuded rings, ACh failed to produce relaxation, and responses did not differ among groups (Figure 7B; Table 1). Furthermore, no significant differences were observed in ACh-induced vascular relaxation in endothelium-intact rings pre-contracted with Phe in the presence of L-NAME (Figure 7C; Table 1), confirming that the observed relaxation responses are dependent on endothelial NO-synthase activity.

**FIGURE 7:**
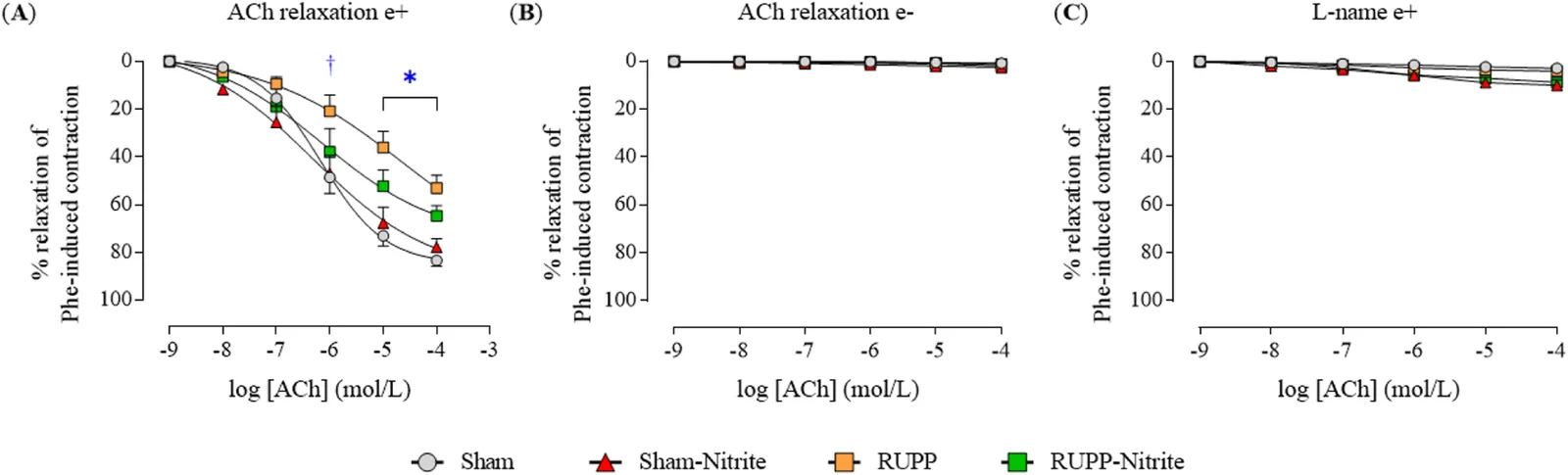
Effects of sodium nitrite treatment on acetylcholine (ACh)-induced vascular relaxation in endothelium-intact **(A)**, endothelium-denuded **(B)**, and endothelium-intact rings in the presence of L-NAME **(C)** of thoracic aortas pre-contracted with phenylephrine (Phe), measured on gestational day 21 in the Sham, Sham-Nitrite, RUPP, and RUPP-Nitrite groups (n = 10 animals per group). Data are expressed as mean ± SEM. Statistically significant differences between groups are indicated by a *post hoc* symbol or a bracket with a *post hoc* symbol at the corresponding concentrations based on two-way ANOVA followed by Tukey’s *post hoc* test. *p < 0.05 for Sham vs. RUPP and RUPP-Nitrite groups, and Sham-Nitrite vs. RUPP group; †p < 0.05 for Sham and Sham-Nitrite vs. RUPP group.

### 3.8 Effects of sodium nitrite on Phe-induced vascular contraction

Significant increases in the contractile vascular response to Phe were observed in endothelium-intact thoracic aortic rings in the RUPP group compared to the Sham-Nitrite group at 10^−8^ and 5.5 × 10^-8^ M (Figure 8A; Table 1). Increases in the contraction were also found in the RUPP group compared to the RUPP-Nitrite group at 5.5 × 10^-8^ M and the RUPP group compared to the Sham, Sham-Nitrite and RUPP-Nitrite groups at 10^−7^, 5.5 × 10^−7^, 10^−6^, 5.5 × 10^−6^, 10^−5^, 5.5 × 10^−5^, and 10^−4^ M (Figure 8A; Table 1).

**FIGURE 8:**
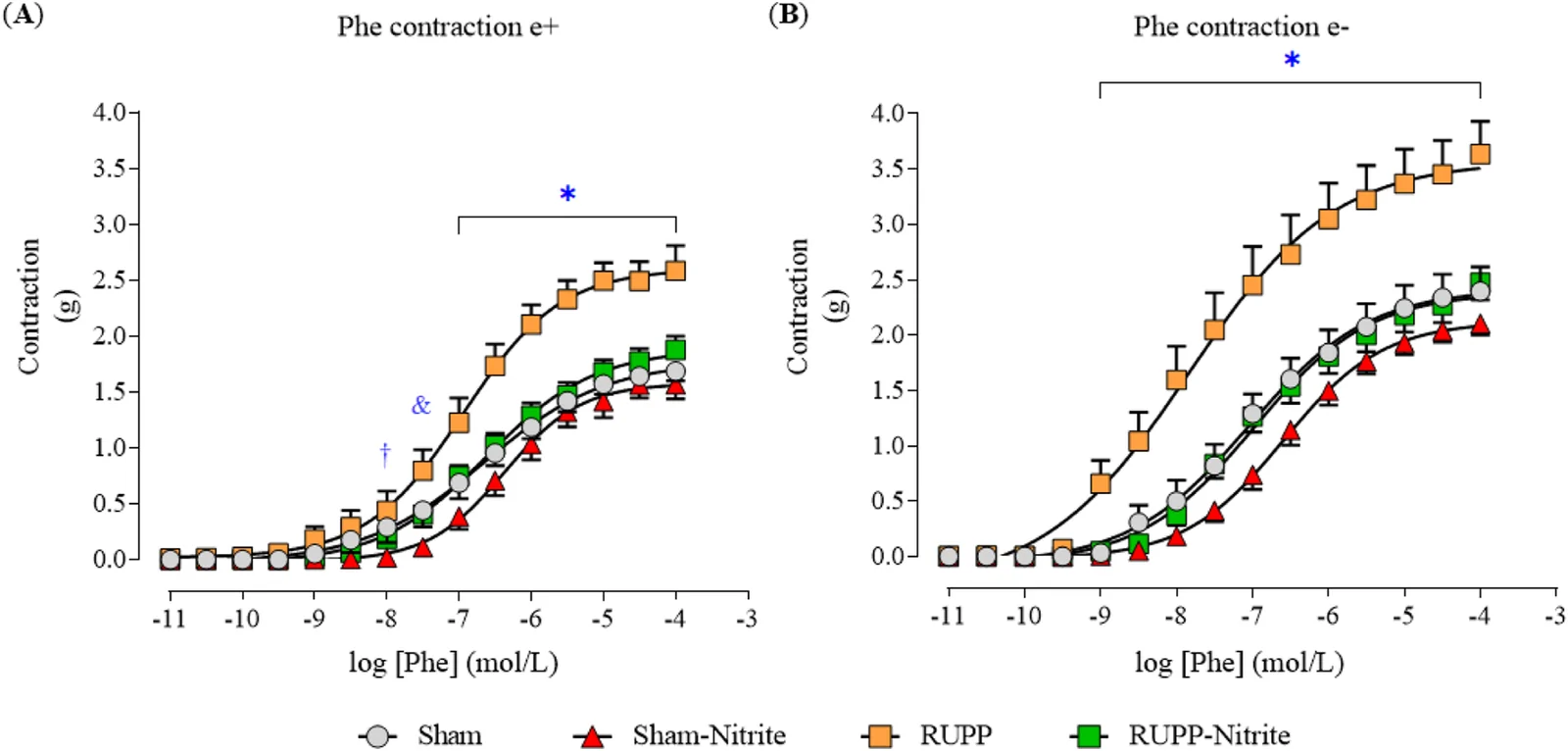
Effects of sodium nitrite treatment on phenylephrine (Phe)-induced vascular contraction in endothelium-intact **(A)** and endothelium-denuded **(B)** thoracic aortic rings measured on gestational day 21 in the Sham, Sham-Nitrite, RUPP, and RUPP-Nitrite groups (n = 10 animals per group). Data are expressed as mean ± SEM. Statistically significant differences between groups are indicated by *post hoc* symbols or a bracket with a *post hoc* symbol at the corresponding concentrations based on two-way ANOVA followed by Tukey’s *post hoc* test. *p < 0.05 for RUPP vs. Sham, Sham-Nitrite, and RUPP-Nitrite groups; †p < 0.05 for Sham-Nitrite vs. RUPP group; & p < 0.05 for RUPP vs. Sham-Nitrite and RUPP-Nitrite groups.

In endothelium-denuded thoracic aortic rings, significant increases in the contractile response to Phe were noted in the RUPP group compared to the Sham, Sham-Nitrite, and RUPP-Nitrite groups in concentrations 10^−9^, 5.5 × 10^−9^, 10^−8^, 5.5 × 10^−8^, 10^−7^, 5.5 × 10^−7^, 10^−6^, 5.5 × 10^−6^, 10^−5^, 5.5 × 10^−5^, and 10^−4^ (Figure 8B; Table 1).

### 3.9 Assessment of SNP-Induced vascular relaxation responses

No significant differences were observed in SNP-induced vascular relaxation among the Sham, Sham-Nitrite, RUPP, and RUPP-Nitrite groups across the concentration range of 10^−11^ to 10^−6^ M in endothelium-intact thoracic aortic rings pre-contracted with Phe (Figure 9A; Table 1). Similarly, in endothelium-denuded rings, SNP-induced vascular relaxation did not differ significantly among the experimental groups across the same concentration range (Figure 9B; Table 1).

**FIGURE 9:**
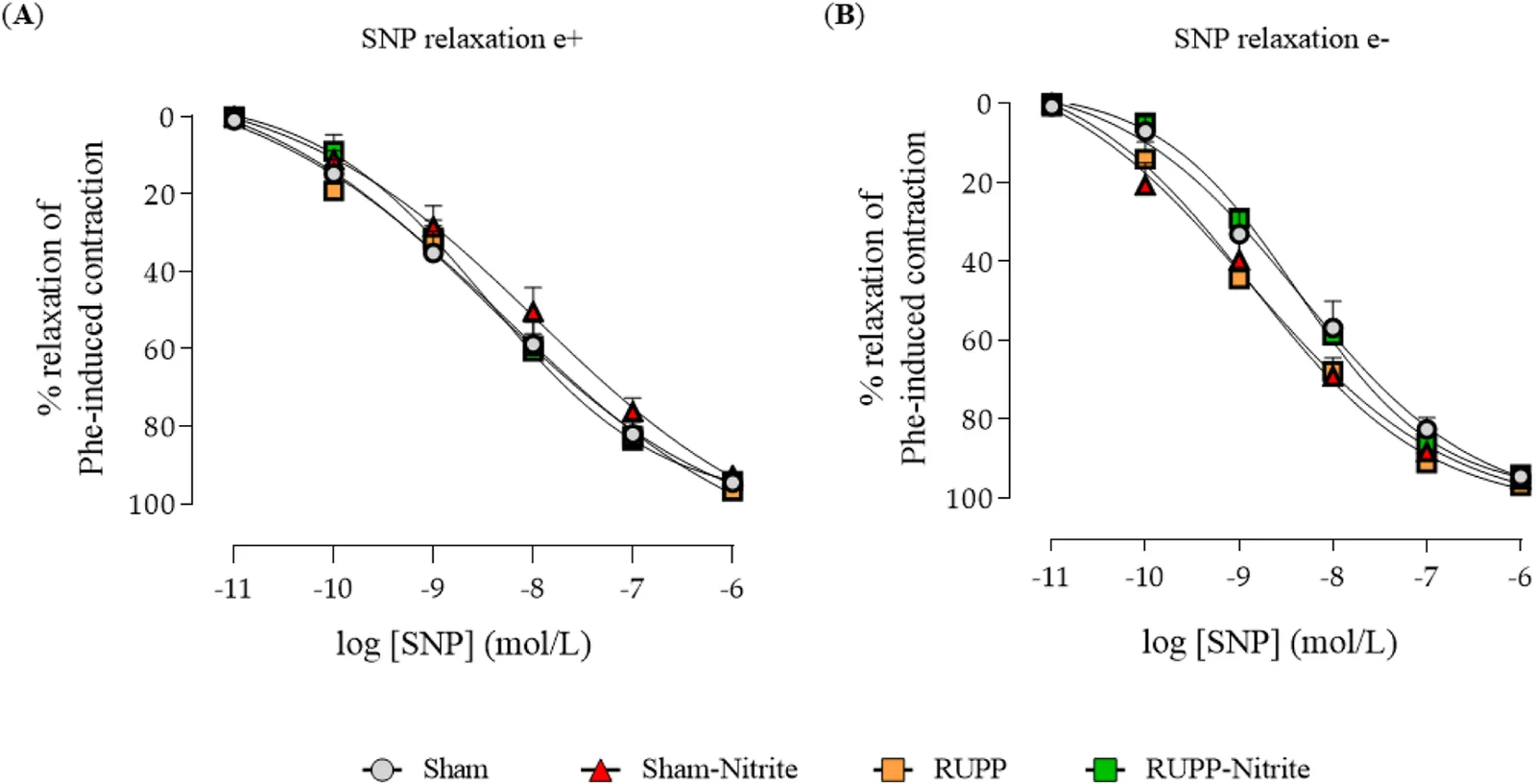
Assessment of sodium nitroprusside (SNP)-induced vascular relaxation responses in endothelium-intact **(A)** and endothelium-denuded **(B)** thoracic aortic rings pre-contracted with phenylephrine (Phe), measured on gestational day 21 in the Sham, Sham-Nitrite, RUPP, and RUPP-Nitrite groups (n = 10 animals per group). Data are expressed as mean ± SEM.

## 4 Discussion

The results of the present study demonstrate that reduced uteroplacental blood perfusion induced a classical PE phenotype characterized by increased blood pressure, vascular endothelial dysfunction, and impaired fetal and placental growth. Additionally, the RUPP model also induced increased ADMA levels. These findings are consistent with the literature describing the RUPP model as one of the most robust experimental models for simulating the pathophysiology of PE, particularly with regard to placental ischemia leading to maternal systemic vascular endothelial dysfunction (Martins et al., 2023; Crews et al., 2000).

The significant increases in systolic, diastolic, and mean arterial pressure observed in the RUPP group support the central role of placental ischemia in the pathogenesis of PE. Reduced uteroplacental blood flow triggers the release of circulating factors that promote vascular endothelial dysfunction and increased peripheral vascular resistance, as previously demonstrated (Granger et al., 2002; Da Silva et al., 2024). Accordingly, autonomic alterations have also been described in PE, contributing to hemodynamic instability [16]. In this context, the attenuation of hemodynamic disturbances in the RUPP group treated with sodium nitrite suggests a relevant antihypertensive effect, probably mediated by the conversion of nitrite to NO under hypoxic conditions, a mechanism previously described as an alternative pathway for NO generation (Lundberg et al., 2008; Martins et al., 2023). However, given that plasma NOx levels were not significantly increased following sodium nitrite treatment, the present findings do not support attributing the antihypertensive effect exclusively to increased systemic NO bioavailability. Instead, both NO-dependent and NO-independent mechanisms may contribute to the vascular effects of nitrite in the RUPP model.

An important finding with potential translational and safety implications was the significant reduction in diastolic and mean arterial pressure observed in Sham-Nitrite animals compared with Sham animals. This observation indicates that the blood pressure-lowering effect of sodium nitrite is not restricted to the hypertensive state induced by RUPP and may also occur in normotensive pregnancy. Although a reduction in blood pressure may be beneficial in the context of pregnancy-associated hypertension, excessive maternal hypotension could compromise uteroplacental perfusion and consequently affect fetal oxygen and nutrient delivery. However, the present study was not designed to determine whether the reduction in blood pressure observed in Sham-Nitrite animals resulted in impaired uteroplacental perfusion, and no evidence of such an effect can be established from the present data.

In addition to the hemodynamic alterations, the reduction in placental and fetal weights observed in the RUPP group indicates impaired intrauterine development, consistent with fetal growth restriction. The literature demonstrates that placental hypoxia compromises nutrient and oxygen supply, directly affecting fetal growth (Li et al., 2012). In the present study, sodium nitrite treatment partially prevented these alterations, reinforcing the effect of nitrite-derived NO in participating actively in vasodilation and placental perfusion, thereby supporting fetal development. Nevertheless, because the present study did not directly assess placental NO generation or distinguish between NO-dependent and NO-independent effects of nitrite, these findings should be interpreted as being consistent with, rather than demonstrating, a role for nitrite-derived NO in these beneficial effects. In support of this suggestion, previous evidence demonstrated enhanced nitrite-mediated relaxation of placental blood vessels exposed to hypoxia in pregnancies complicated by fetal growth restriction (Tropea et al., 2021).

Significant increases in ADMA levels were also observed in the RUPP group of the present study. ADMA is an endogenous inhibitor of eNOS, which has been recognized as an important marker of endothelial dysfunction (Dymara-Konopka and Laskowska, 2019). Increased ADMA contributes to reducing NO bioavailability, resulting in marked vasoconstriction and increased blood pressure (Leone et al., 1992), which corroborates the results of the present study. These findings are consistent with studies demonstrating an imbalance between NO production and degradation in PE (Ehsanipoor et al., 2013). Interestingly, the present findings demonstrate that sodium nitrite treatment attenuates the RUPP-induced increase in ADMA levels. Hence, our current results are in accordance with the notion that the balance on the ADMA-NO axis plays a fundamental role in regulating vascular tone during pregnancy, being responsible for the adaptive vasodilation required to increase uteroplacental blood flow (Sutton et al., 2020). Therefore, increased ADMA and reduced NO availability may directly contribute to increased vascular resistance and to the vascular endothelial dysfunction observed in PE (Sutton et al., 2020). Importantly, although the reduction in ADMA observed following sodium nitrite treatment occurred in parallel with improvements in hemodynamic and vascular parameters, the present study does not establish a causal relationship between ADMA reduction and increased NO bioavailability. The absence of a significant increase in plasma NOx after nitrite treatment further suggests that the reduction in ADMA cannot be attributed solely to an increase in circulating NOx and may involve additional NO-independent or redox-sensitive mechanisms.

The present vascular reactivity findings further support the PE phenotype. The impaired ACh-induced relaxation observed in the RUPP group indicates endothelial dysfunction, since this response depends on the integrity of the eNOS-NO pathway. Studies using experimental models of PE have demonstrated reduced endothelium-dependent vasodilation associated with decreased NO bioavailability (Da Silva et al., 2024; Walsh et al., 2009). The partial improvement in ACh-induced vascular relaxation observed in the RUPP-Nitrite group suggests partial restoration of NO-dependent endothelial function. Moreover, the increased contractile response to Phe observed in the RUPP group indicates vascular hyper-reactivity, a feature frequently associated with PE. This phenomenon may be explained by the reduction in the modulatory effect of NO on vascular smooth muscle tone, leading to a predominance of vasoconstrictor stimuli (Martins et al., 2023). The attenuation of Phe-induced vascular hyper-reactivity in the RUPP-Nitrite group highlights the role of nitrite as an alternative source of NO, particularly under RUPP-induced hypoxic conditions. This mechanism suggests that nitrite-derived NO may counteract the contractile response induced by Phe. The present findings are consistent with previous studies demonstrating that orally administered sodium nitrite prevented the increased Phe-induced vasoconstriction in the two-kidney/one-clip (2K1C) renovascular hypertension model in rats (Oliveira-Paula et al., 2023). Additionally, the lack of significant differences in SNP-induced relaxation among the groups, in both endothelium-intact and endothelium-denuded aortic rings, indicates that endothelium-independent NO-induced vasorelaxation was not affected by either RUPP or sodium nitrite treatment. These findings further suggest that the vascular alterations observed in response to ACh are primarily related to endothelial dysfunction rather than to changes in the responsiveness of vascular smooth muscle to NO.

The results of the present study support the evidence that increased ADMA levels, reduced NO bioavailability, and endothelial dysfunction are central events in the pathophysiology of PE (Dymara-Konopka and Laskowska, 2019). Importantly, the present findings demonstrate that sodium nitrite treatment effectively attenuated hemodynamic disturbances, improved fetal and placental parameters, partially restored endothelial and vascular functions, and attenuated the RUPP-induced increase in ADMA levels. These effects observed in the RUPP-Nitrite group may be attributed to the conversion of nitrite into NO under hypoxic conditions, a mechanism particularly relevant in the context of placental ischemia (Martins et al., 2023; Da Silva et al., 2024). In this regard, although the mechanisms underlying the reduction in circulating ADMA following sodium nitrite treatment remain unclear, recent evidence *in vitro* has shown that NO can reduce the ADMA generation (Taniguchi et al., 2024). However, these mechanistic assays were not assessed in the present study, and this potential mechanism remains to be experimentally established. Further studies are therefore warranted to determine whether modulation of the enzymes involved in ADMA synthesis contributes to the reduction in circulating ADMA induced by sodium nitrite in experimental PE.

Previous evidence has demonstrated that nitrite may also restore endothelial function and reduce vascular hyperreactivity to vasoconstrictors, improving hemodynamics regardless of NO metabolite levels. In this regard, it has been suggested that nitrite may induce sustained hypotensive effects through an NO-independent including redox-based pathway. In this pathway, nitrite generates reactive species like hydrogen peroxide, leading directly to vascular relaxation (Feelisch et al., 2019), and thereby, explaining in part the improvements in blood pressure despite not having significant increases in NOx in the RUPP + Nitrite group. Thus, the present findings may be interpreted within a framework in which both NO-dependent and NO-independent pathways contribute to the effects of sodium nitrite. The lack of a significant increase in plasma NOx following treatment favors caution against attributing the observed reduction in blood pressure exclusively to increased systemic NO availability, while the vascular findings remain compatible with a contribution of NO-related signaling at the tissue level that may not be reflected by circulating NOx measurements. Accordingly, the relative contribution of NO-dependent versus NO-independent mechanisms to the antihypertensive and ADMA-lowering effects of nitrite remains to be determined.

To the best of our knowledge, the present study is the first to provide evidence that sodium nitrite treatment attenuates RUPP-induced increases in ADMA levels. These findings suggest that sodium nitrite-derived NO may emerge as a potential therapeutic strategy to mitigate the hemodynamic and vascular endothelial disturbances associated with PE by directly targeting the ADMA-NO axis, one of the main molecular pathways regulating endothelial function that is impaired in PE. The ADMA-NO axis is considered a fundamental mechanism in the regulation of vascular tone, where an imbalance among its components leads to endothelial dysfunction (Boeger, 2003), including in PE (Dymara-Konopka and Laskowska, 2019). The present results justify future studies aimed at optimizing nitrite-based therapy for PE-related vascular endothelial dysfunction and other conditions associated with increased ADMA levels and deficient NO bioavailability. However, the translation of these findings into clinical practice requires additional investigations to evaluate the safety, dose–response relationship, and efficacy of sodium nitrite treatment in humans, since the selected dose of sodium nitrite (15 mg/kg/day; 0.217 mmol/kg) was based on rodent models of hypertension (Amaral et al., 2015; Gonçalves-Rizzi et al., 2016), which is higher than standard clinical doses (Hughan et al., 2020), and plasma nitrite or nitrate concentrations were not directly measured to confirm target engagement. While the lack of these measurements represents a study limitation, this dosage strictly aligns with established experimental protocols (Amaral et al., 2015; Gonçalves-Rizzi et al., 2016). In this regard, a translational dose gap remains as rodents possess higher metabolic rates and accelerated pharmacokinetics for NO-releasing agents compared to humans.

Some limitations of the present study should be acknowledged, and the findings should therefore be interpreted with caution. While multiple comparison corrections were applied within each ANOVA dataset, outcome variables were analyzed independently without global adjustments across the entire array of parameters. Furthermore, although sodium nitrite treatment in RUPP rats reduced circulating ADMA levels, the underlying mechanisms remain to be elucidated. Additionally, plasma ADMA was quantified using a commercial ELISA assay, which may have lower specificity and accuracy than reference analytical methods such as Liquid Chromatography-Tandem Mass Spectrometry (LC-MS/MS) or High-Performance Liquid Chromatography (HPLC). Thus, future studies using these reference methods are warranted to independently validate and confirm the present ADMA findings. Moreover, vascular reactivity was assessed in thoracic aortic rings, a large conduit vessel, rather than in resistance vessels such as the uterine arteries. Hence, the vascular effects observed in the aorta may not fully reflect those in the uteroplacental circulation, and further studies are needed to assess the effects of sodium nitrite on resistance vascular beds. In addition, although the present study demonstrated functional changes in vascular reactivity, no histological or structural assessment of the vascular endothelium was performed. Accordingly, structural alterations in endothelial integrity underlying the observed functional responses could not be directly evaluated. Future studies incorporating histological and structural analyses of the vascular endothelium will be important to complement the functional findings and determine whether sodium nitrite-induced changes in vascular reactivity are accompanied by structural modifications. Although nitrite treatment improved fetal outcomes, the present study does not allow us to determine whether these effects resulted directly from nitrite-derived NO or occurred secondarily to the attenuation of maternal hypertension. Additional studies are also needed to evaluate the dose-response relationship and potential toxicity of sodium nitrite in pregnant rats and preeclamptic-like rats. Therefore, further mechanistic investigations are required to clarify the pathways involved, establish causality, and distinguish direct fetal effects from those mediated by improvements in maternal hemodynamics.

## 5 Conclusion

The findings of the present study demonstrate that reduced uteroplacental blood perfusion induces a phenotype consistent with PE, characterized by hypertension, impaired fetal and placental development, vascular endothelial dysfunction, and imbalance in the ADMA–NO axis. Sodium nitrite treatment was able to attenuate these disorder outcomes in the experimental PE model. Thus, the present results reinforce the role of increased ADMA levels in the pathophysiology of PE and indicate sodium nitrite as a potential therapeutic approach.

## Data Availability

The original contributions presented in the study are included in the article/supplementary material, further inquiries can be directed to the corresponding author.
